# Machine and Deep Learning for the Diagnosis, Prognosis, and Treatment of Cervical Cancer: A Scoping Review

**DOI:** 10.3390/diagnostics15121543

**Published:** 2025-06-17

**Authors:** Blanca Vazquez, Mariano Rojas-García, Jocelyn Isabel Rodríguez-Esquivel, Janeth Marquez-Acosta, Carlos E. Aranda-Flores, Lucely del Carmen Cetina-Pérez, Susana Soto-López, Jesús A. Estévez-García, Margarita Bahena-Román, Vicente Madrid-Marina, Kirvis Torres-Poveda

**Affiliations:** 1 Unidad Académica del Instituto de Investigaciones en Matemáticas Aplicadas y en Sistemas del Estado de Yucatán, Universidad Nacional Autónoma de México (UNAM), Mérida 97357, Mexico; 2 Department of Animal Sciences, Facultad de Estudios Superiores Cuautitlán, Universidad Nacional Autónoma de México (UNAM), Cuautitlán Izcalli 54714, Mexico; mariano.rojas@cuautitlan.unam.mx; 3 Center for Research on Infectious Diseases, Instituto Nacional de Salud Pública (INSP), Cuernavaca 62100, Mexico; joce.rdz.esq@gmail.com (J.I.R.-E.); mbahena@insp.mx (M.B.-R.); vmarina@insp.mx (V.M.-M.); kjtorres@insp.mx (K.T.-P.); 4 Colposcopy Department, Luis Catelazo Ayala Hospital, Instituto Mexicano del Seguro Social (IMSS), Mexico City 01090, Mexico; drajanmarac@gmail.com; 5 Oncology Department, Hospital General de Mexico Eduardo Liceaga, Mexico City 06720, Mexico; aranda_floresc@hotmail.com; 6 Department of Clinical Research and Medical Oncology, Instituto Nacional de Cancerología (INCAN), Mexico City 14080, Mexico; lucelycetina.incan@gmail.com; 7 Femme Vite Center, Cuernavaca 62374, Mexico; susysoto25@exalumno.unam.mx; 8 Center for Population Health Research, Instituto Nacional de Salud Pública (INSP), Cuernavaca 62100, Mexico; jesus.alejandro@insp.edu.mx; 9 Secretaría de Ciencia, Humanidades, Tecnología e Innovación (SECIHTI), Mexico City 03940, Mexico

**Keywords:** uterine cervical neoplasms, machine learning, deep learning, diagnosis, prognosis, therapeutics

## Abstract

**Background/Objectives:** Cervical cancer (CC) is the fourth most common cancer among women worldwide. This study explored the use of machine learning (ML) and deep learning (DL) in the prediction, diagnosis, and prognosis of CC. **Methods:** An electronic search was conducted in the PubMed, IEEE, Web of Science, and Scopus databases from January 2015 to April 2025 using the search terms ML, DL, and uterine cervical neoplasms. A total of 153 studies were selected in this review. A comprehensive summary of the available evidence was compiled. **Results:** We found that 54.9% of the studies addressed the application of ML and DL in CC for diagnostic purposes, followed by prognosis (22.9%) and an incipient focus on CC treatment (22.2%). The five countries where most ML and DL applications have been generated are China, the United States, India, Republic of Korea, and Japan. Of these studies, 48.4% proposed a DL-based approach, and the most frequent input data used to train the models on CC were images. **Conclusions:** Although there are results indicating a promising application of these artificial intelligence approaches in oncology clinical practice, further evidence of their validity and reproducibility is required for their use in early detection, prognosis, and therapeutic management of CC.

## 1. Introduction

Cervical cancer (CC) is the fourth most common cancer among women worldwide, with an age-standardized incidence rate of 14.1 cases per 100,000 women-years, and the third leading cause of mortality due to cancer in women, with a mortality rate of 7.1 deaths per 100,000 women-years [1].

Regions with the highest CC disease burden include sub-Saharan Africa, Latin America, and Asia [2]. CC is primarily caused by a persistent infection of high-risk Human Papilloma Virus (HR-HPV). In 2020, the World Health Assembly established a strategy for the elimination of CC [3], which comprises prevention (90% of girls aged 15 years or younger fully vaccinated against HPV), early detection (70% of women aged 35–45 years screened by molecular methods to detect HR-HPV DNA), and guaranteed treatment of women (90% of women) diagnosed with CC. Many advances have been made to improve the technologies available for the early diagnosis of CC [4], its prognosis [5], and the treatment of precancerous lesions and CC [6]; new technologies have emerged for faster and more accurate diagnosis and improved prognosis prediction.

In this regard, machine learning (ML) and deep learning (DL) are two approaches from artificial intelligence (AI) that have contributed to clinical practice [7,8,9] in the development of tools to support the tasks of diagnosis, prognosis, and treatment of various cancers [10,11,12,13,14,15]. ML is a subfield of artificial intelligence (AI) that focuses on using data and algorithms to give computers the ability to learn without explicitly being programmed [16]. In contrast, DL is a specific subfield of ML that leverages artificial neural networks to mimic the learning process of the human brain [17]. To learn, both ML and DL algorithms require input data that can be images, text, or signals that, through an iterative process, identify patterns in this data and produce the desired result.

ML algorithms can be categorized into supervised and unsupervised learning depending whether the data are labeled or unlabeled [18]. In supervised learning, the input data used during training are labeled; this means that the algorithm models relationships and dependencies between the label and the input features. Moreover, the model output prediction can be compared with the label; if the output is incorrect, this can be corrected during training [19]. In supervised learning, the goal is to learn a mapping *f* from inputs x∈X to outputs y∈Y, where the inputs *x* are called features or predictors, which is often a fixed-dimensional vector of numbers, such as a person’s age and weight, or the pixels in an image [20]. The output *y* is called a label or response.

Classification and regression are the most common tasks in supervised learning, while clustering and association are tasks in unsupervised learning. Classification is a type of supervised learning that is used to predict categorical values, for instance, given a set of a patient’s clinical variables, to predict whether they have COVID-19 or not (binary classification) [21] or to categorize a cervical cell image into three classes: normal, low-grade intraepithelial lesion, or high-grade intraepithelial lesion (multiclass classification) [22]. In contrast, regression is used to predict continuous values, such as the price of a car given a set of features or the predicted dose in brachytherapy for CC [23]. The most common algorithms for classification and regression are logistic regression (LR), support vector machine (SVM), random forest (RF), decision trees (DT), and naive Bayes (NB) [9].

In contrast, in unsupervised learning, the input data used are unlabeled; this means that there is no guide that indicates the model’s output. That is, the models just observed a set of inputs *x* without any corresponding outputs *y*. The idea of unsupervised learning is to discover patterns, relationships, similarities, and differences in the data without any explicit guidance [20]. Clustering is a type of unsupervised learning that is used to find patterns and split data into different groups with common features, such as grouping cells based on their characteristics to model the degrees of abnormality in CC [24]. The most common algorithms for clustering are principal component analysis (PCA), density-based spatial clustering of applications with noise (DBSCAN), and K-means clustering [25].

On the other hand, neural networks, which are the core of DL, are layers stacked on top of each other, where each layer is composed of a set of neurons, which are computing units that attempt to mimic the behavior of a biological neuron [7,17]. The term ‘neuron’ appeared in 1943, proposed by McCulloch and Pitts when they mathematically formalized a neuron’s behavior and studied its implications for computing and processing information. This mathematical model has inspired the development of hundreds of different models based on neuronal networks. The most common models are convolutional neural networks (CNNs) [26], Transformer [27], and residual networks [28].

In recent years, the amount of research in the clinical area using ML and DL models has increased exponentially because these models can help in clinical decision-making, such as early warning, individualization of treatment, and improvement of progress in clinical trials [8,29,30]. In particular, several works have emerged in the study of cervical neoplasm using ML and DL models due to immense interest in the field [5,31,32]. Therefore, we performed a scoping review [33] to map the body of literature on the use of these models for the diagnosis, prognosis, and treatment of CC in order to provide updated evidence on the application of ML and DL in CC, assess these approaches’ potential scope in oncological clinical practice, and encourage their adoption in clinical decision-making support, especially in contexts where qualified personnel are lacking.

## 2. Materials and Methods

### 2.1. Search Strategy

**Figure 1:**
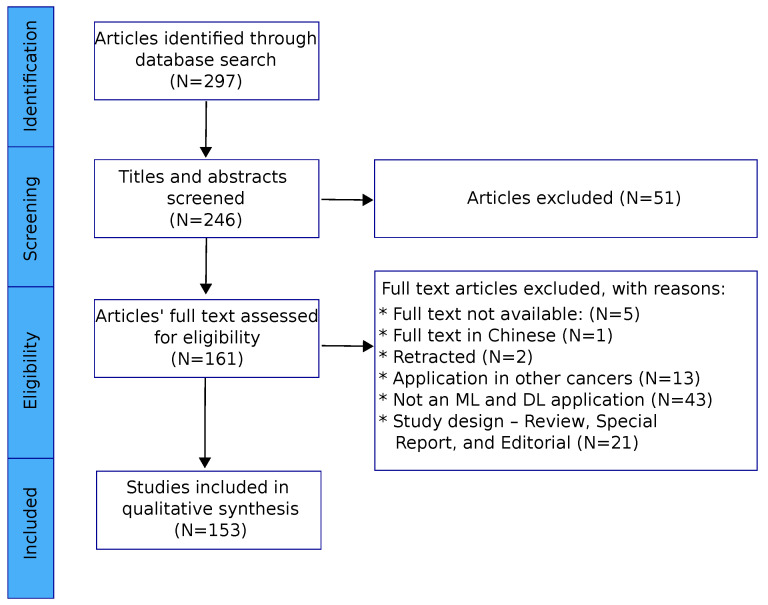
Flowchart of the search procedure following PRISMA-ScR (PRISMA Extension for Scoping Reviews).

The review’s population, concept, context (PCC) question was, “What uses have machine learning and deep learning had according to the evidence found in the diagnosis, prognosis, and treatment of cervical cancer?”, in which P = cervical cancer, C = proposed use, and C = machine and deep learning. To address this question, we conducted a scoping review following the PRISMA-ScR statement recommendations (Supplementary Table S1) [33]. There is no registered review protocol as this review is a scoping review. A search in the MEDLINE database was performed through the PubMEd database browser, Scopus, Web Of Science, and IEEE, with a text formed by combining the following terms: “Machine Learning”, “Uterine Cervical Neoplasms”, “Diagnosis”, and “Prognosis”. The Boolean operator (“AND”) was used to link the search terms with the research question. The search strategy applied to locate studies in the main electronic database was as follows: ((machine learning[MeSH Terms]) AND (uterine cervical neoplasm[MeSH Terms])). The scope of the bibliographic search was expanded based on the reference lists of the retrieved articles. Figure 1 summarizes the flow diagram of the search process followed in this review.

### 2.2. Study Selection

All searched articles were assessed to determine their eligibility for inclusion in the review. First, the studies were assessed by reading their titles/abstracts. Papers considered relevant after this initial review were selected for further evaluation through full text reading. Four authors manually screened the selection of the original articles, and one of them acted as a content reviewer, being a specialist in the area. After evaluation of the full text, 153 articles were deemed eligible and included in this scoping review.

### 2.3. Eligibility Criteria

All the primary studies retrieved were reevaluated to assess whether they met the inclusion criteria for the scoping review, as outlined below.

### 2.4. Study Design

Cross-sectional and case–control studies, cohort studies, and clinical assays with original data reporting the use of ML and DL for the prediction, diagnosis, and prognosis of CC.

### 2.5. Language

The search was limited to studies published in English.

### 2.6. Publication Date

Publications available online from January 2015 to April 2025 were considered. The evidence included in this scoping review was predominantly within the past decade, the period during which machine and deep learning have been most widely applied in CC research.

### 2.7. Exclusion Criteria

During the full-text assessment of the articles, we evaluated the study design, study model, research settings, paper quality, and relevant outcomes. Review, special report, and editorial papers; papers with different types of cancers as outcomes; papers without machine and deep learning applications; and papers with a low quality score were omitted.

### 2.8. Data Extraction

Four authors independently gathered all pertinent data using a data extraction template created in Microsoft Excel (Microsoft Co., Redmond, WA, USA). The extraction template included details such as the name of the first author of each study, publication year, reference, study type, study model, study design, aim of the study, medical use, sample group, source population, age group, data source, type of patient, clinical specialist participation, process in which the clinical specialist participated, database, multicenter study, ML and DL algorithm implemented with the best performance, the best performance metrics, data augmentation technique, cross validation technique, external validation, study’s limitations, available code, available data, available app/web application, and criteria to evaluate the study’s quality. Each section was filled out in a single column, with rows containing data from each primary study. Any discrepancies during the data extraction process were identified and addressed through discussion.

### 2.9. Outcome Measurement

Any use of ML and DL for the diagnosis, prognosis, and treatment of CC was analyzed and treated as an outcome.

### 2.10. Quality Assessment

The quality of the primary studies included in this scoping review was evaluated using the Strengthening the Reporting of Observational Studies in Epidemiology (STROBE) scale, designed for assessing the quality of observational studies [34]. To evaluate the quality of a study, an 11-item scale based on the STROBE principles was created through consensus among the 11 authors. Each item was categorized according to the introduction, methods, results, and discussion sections. The items addressed aspects such as study design, recruitment, participant description, and overall quality. The score was represented in arbitrary units (a.u.) ranging from 0 to 23 (from lower to higher quality). The authors assessed the quality independently, and any disagreements were resolved after discussion. The quality analysis used in the scoping review is described in Table 1.

### 2.11. Synthesis of Results

The research team reviewed and discussed the potential applications of ML or DL for the diagnosis, prognosis, and treatment of cervical neoplasm based on the data included. Descriptive analyses were provided as narrative summaries, given the heterogeneity of the literature. A narrative summary involves presenting findings in a straightforward way [35].

### 2.12. Statistical Analysis

The data were gathered into a single spreadsheet and imported into Microsoft Excel 2019 (Microsoft Co., Redmond, WA, USA) for validation and coding. Fields that allowed string values were checked for unrealistic entries. The data were subsequently exported to STATA version 16 (StataCorp, College Station, TX, USA) for analysis. Descriptive statistics were used to summarize the data, and frequencies and percentages were calculated to summarize the data. Frequencies and percentages were utilized to describe nominal data.

## 3. Results

### 3.1. Included Studies

Our electronic search retrieved 297 study records. After title and abstract screening, 246 articles were retained for full-text review. Of these, 93 were excluded, resulting in a total of 153 unique articles included in our scoping review. These research articles were published between January 2015 and April 2025.

### 3.2. Quality Assessment of Included Studies

The mean (±SD) quality of the 153 selected observational studies was 15 (±0.6) a.u. (range 10 to 23), indicating a satisfactory level. Table 2 describes the quality assessment of individual studies using the STROBE scale.

### 3.3. Clinical Applications of ML and DL in Cervical Cancer

**Figure 2:**
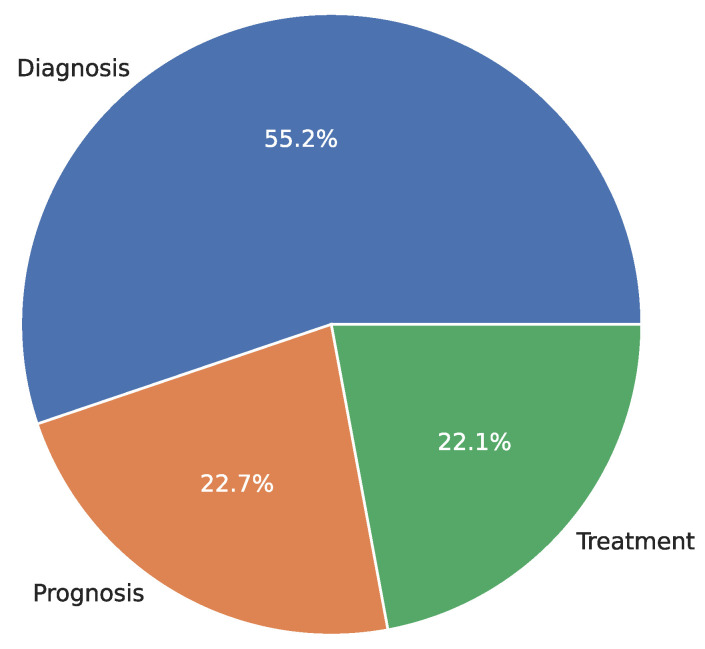
Distribution of the selected articles per clinical applications using ML and DL for CC.

Figure 2 summarizes the clinical applications found in the reviewed literature grouped as follows: diagnosis (54.9%), prognosis (22.9%), and treatments (22.2%). For diagnostic applications, we found that most studies addressed the development of models to diagnose lesions in colposcopy images (e.g., [75,81,133]); we also grouped the studies that addressed diagnosis into six main targets: (i) states of CC (32.02%) [74,75,81,93], (ii) screening for CC (20.26%) [39,41,43], (iii) recurrence (cancer progression) (0.65%) [62], (iv) type prediction of Human Papilloma Virus (HPV) (0.65%) [54], (v) cancer progression (0.65%) [62], and (vi) segmentation of targets and organs at risk (OARs) (0.65%) [132].

**Figure 3:**
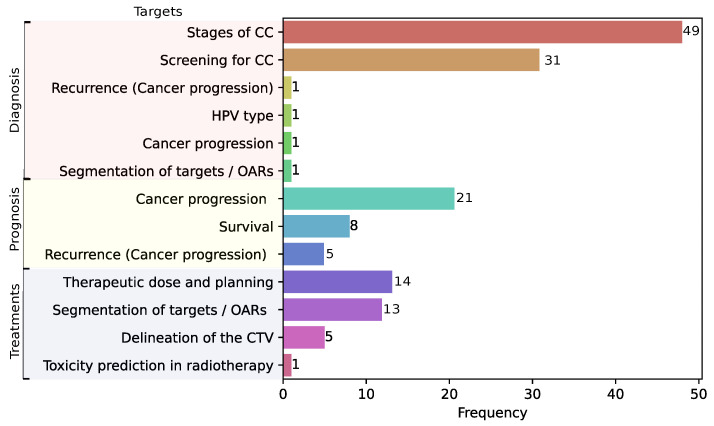
Distribution of targets grouped by clinical applications. The targets appear in order of highest to lowest frequency by clinical application.

For prognosis application, most studies focused on predicting cancer progression using CT and MRI (e.g., [77,78,156]); in particular, three main targets were identified in prognosis: (i) cancer progression (13.72%) [56,61,84], (ii) survival prediction (5.22%) [55,58,98], and (iii) recurrence (3.26%) [176] (e.g., [77,137,178]). For treatment applications, most of the studies focused on automatic segmentation of images to predict doses of treatments using CT (e.g., [68,131,141]). In this clinical application, four main targets were found: (i) therapeutic dose and planning (9.15%) [118,144,146], (ii) segmentation of targets/OARS (8.49%) [109,130,140], (iii) delineation of the clinical target volume (CTV) (3.26%) [115,141,153], and (iv) toxicity prediction in radiotherapy (0.65%) [101]. Figure 3 shows the distribution of targets for each clinical application based on ML and DL in CC.

### 3.4. Prediction Tasks in CC

**Figure 4:**
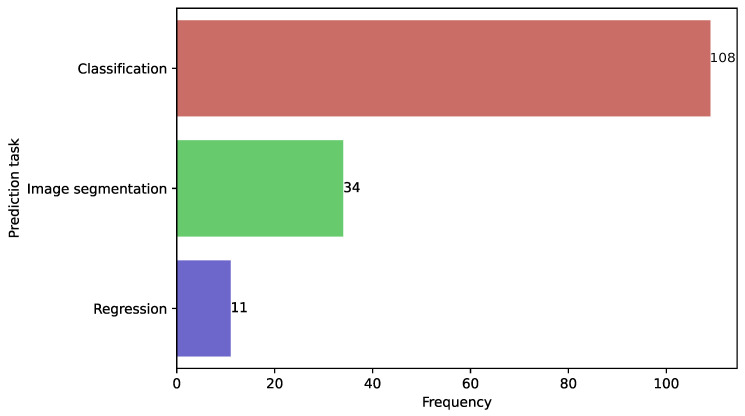
Distribution of the selected articles per prediction task in CC.

Figure 4 displays the frequency of ML and DL objectives in the present selection study. Classification tasks were the most frequently employed, appearing in 108 studies (70.77%), followed by image classification in 34 articles (22.07%); 11 articles employed regression tasks (7.14%). For instance, one study [65] used DL classification to diagnose CC from magnetic resonance imaging (MRI) processing, the results of which were evaluated by experienced radiologists. After evaluation, the authors concluded that the performance of their algorithm was equivalent to that of experienced radiologists. Another study [64] trained DL models for automatic segmentation of OARS from computed tomography (CT) scans. The authors remarked that their algorithm is useful for automatic dose optimization for advanced radiation therapy strategies. Among the regression tasks, one study [98] developed an ML algorithm in the prediction of survival with CC from analyzing patient demographics, vital signs, laboratory test results, tumor characteristics, and treatment types. The authors concluded that their algorithm achieved a superior performance compared with a Cox proportional hazard regression model.

### 3.5. Models Trained in Classification, Segmentation, and Regression Tasks

**Figure 5:**
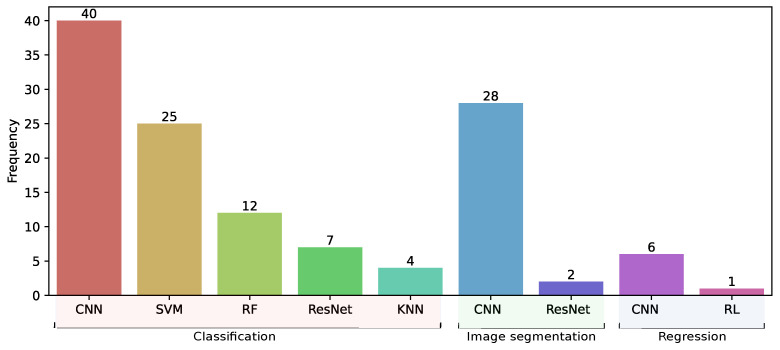
Distribution of the most common ML and DL models used per prediction task.

Figure 5 presents the frequency of models trained using prediction task. Convolutional neural networks (CNNs) were the most frequently employed in the three prediction tasks analyzed. For classification tasks, CNN was the most used, with 40 articles [40,41,54], followed by support vector machine (SVM) [78,104,105], random forest (RF) [94,165,174], ResNet [52,53,72], and k-nearest neighbors (KNN) [81,138]. For instance, Liu et al. [41] described that an algorithm for cytopathology cell image classification based on CNN indicated that their algorithm was robust to changes in the aspect ratio of cells in cervical cytopathological images. Similarly, we found that CNN-based models were the most commonly used in image segmentation tasks, with 28 identified articles. For this task, Yoganathan et al. [32] presented an algorithm based on CNN for automatic segmentation of targets and OARs in MRI, achieving high performance for the automatic contouring, which could be useful in brachytherapy for CC. Finally, CNN-based models were the most common for regression tasks, with six articles. In this case, Yuan et al. [37] proposed a CNN algorithm for predicting doses in intensity-modulated radiation therapy (IMRT) plans. They concluded that there were no significant differences in dose parameters between automatic and real clinic plans.

### 3.6. Temporal Analysis of ML and DL Implemented in CC

**Figure 6:**
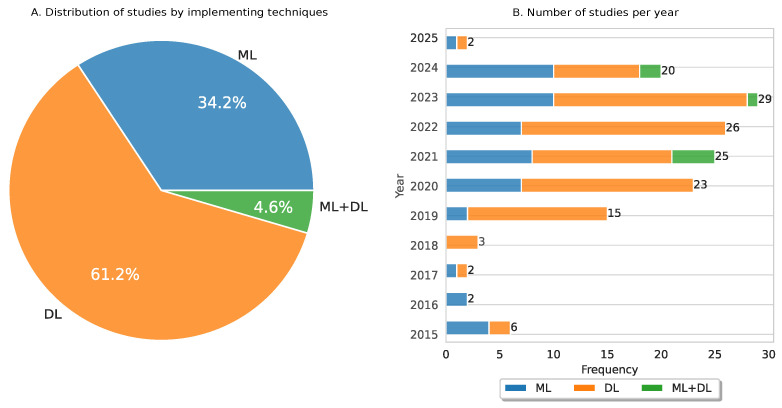
Distribution of studies grouped by ML, DL, and the fusion of ML and DL: (**A**) visualizes the distribution of studies according to implementation techniques; (**B**) lists the number of articles published per year.

Figure 6 presents the distribution of models grouped by technique: ML, DL, and the fusion of ML and DL. Analysis of the 153 reviewed studies showed that the most common implemented technique was DL, with 61.2% (93 studies), whereas ML was implemented in 34.2% (53 studies), and the fusion of ML and DL was used in 4.6% (7 studies). In particular, fusion involves training models with different modalities separately (e.g., images, text, time series). After this training, the goal is to integrate the outputs of these models to improve performance and accuracy in the prediction task. For instance, Mathivanan et al. [160] proposed a hybrid methodology for feature extraction in cytology images based on DL, which was transferred to ML models for CC classification to obtain more accurate and timely interventions.

Figure 6B describes the number of studies by year of publication. In particular, we observed that 2019 to 2024 had the highest number of publications (138 studies) compared to 2015 to 2018 (13 studies). We also identified that 2023 had the largest number of studies (with a total of 29) compared to the other years. In general, it is observed that the number of articles published using DL increases every year. Moreover, we noticed an increase in studies that merge DL and ML techniques in CC prediction.

### 3.7. Analysis of ML-Based Models Implemented in CC

A comparison table for CC prediction with ML techniques is given in Table 3. We found that in most studies, the accuracy was the metric most used to measure the performance of the models; we noted accuracies over 90% in most classification tasks [148,170]. In contrast, for image segmentation, the Dice similarity coefficient (DSC) was used to measure the similarity between two sets or samples. This metric is commonly used in image analysis, particularly for evaluating the performance of image segmentation models.

We also analyzed the validation strategies reported in the literature, namely, cross-validation (CV) with the following: (i) 3 folds, (ii) 5 folds, (iii) 10 folds, and (iv) leave-one-out cross-validation (LOOCV). From 53 studies that used ML models, 19 studies used 10 folds (35.84%), 12 studies used 5 folds (22.64%), and 2 studies used 3 folds and LOOCV, representing 3.77%. We also noted that 33.96% of the studies did not report any validation strategy. Finally, we noticed that of all the articles with ML (53), only 5 studies (9.43%) performed external validation (e.g., [113,166,172]).

### 3.8. Analysis of DL-Based Models Implemented in CC

Table 4 presents a summary of the studies that used DL models for CC prediction. Related to performance, we observed a variety of metrics used, such as (i) accuracy, sensitivity, F1 score, precision, and area under the ROC curve (AUC) for classification tasks, (ii) DSC, Jaccard similarity, and accuracy for image segmentation, and (iii) mean absolute error (MAE), dose volume, and DSC for regression tasks. On the other hand, the most common validation strategy was 5 folds in 19 studies (20.43%), followed by 10 folds in 6 studies (6.45%). We also observed that 60 studies (64.51%) did not report any strategy of validation. Moreover, 13 studies (13.97%) did an external validation to evaluate the performance of their models (e.g., [116,118,153]).

### 3.9. Analysis of Fusion of ML and DL Models Implemented in CC

Table 5 describes a comparison of studies that used ML and DL for CC. For classification tasks, we found the integration of ResNet (DL) with logistic regression (ML) [160], CNN (DL) with SVM (ML) [49], and recurrent neural networks (RNN—DL) with SVM [169], whereas ResNet with SVM [184] and U–Net with SVM [48] were identified by image segmentation. In all the studies in this group, the accuracy was used to measure the algorithm performance, and the validation strategy most used was 5 folds. We also did not find studies that evaluated their models with external data.

### 3.10. Evaluation Metrics for ML and DL in CC

**Figure 7:**
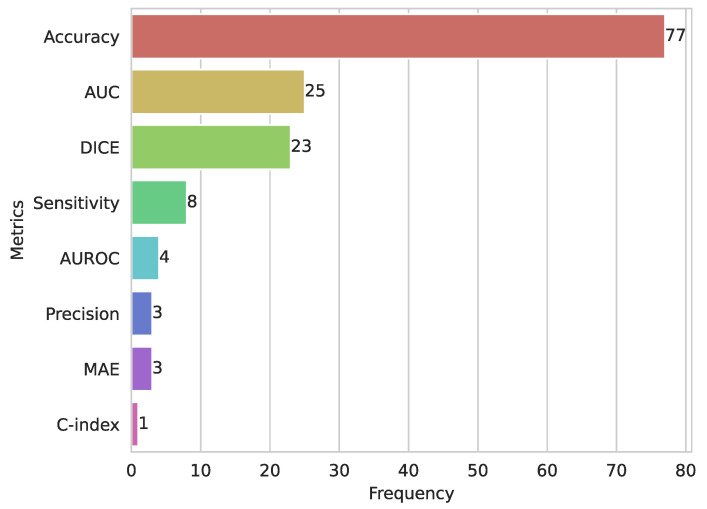
Distribution of the most common metrics used to measure model performance. AUC: area under the curve; DICE: Dice similarity coefficient; AUROC: area under the receiver operating characteristic curve; MAE: mean absolute error.

Figure 7 presents the metrics used to evaluate the models of the selected studies. The accuracy metric was the most used, in 77 studies (50.32%), followed by area under the curve ROC (AUC) in 25 studies (16.33%), and DICE in 23 studies (15.03%). The DICE metric was used in the image segmentation task, while accuracy was used in classification tasks. Other less common metrics were precision (2.6%), mean absolute error—MAE (1.96%), and C–index with 0.65%. In particular, each study used a variety of metrics to measure their model performance in classification, image segmentation, and regression tasks using ML and DL.

### 3.11. Explainability

**Figure 8:**
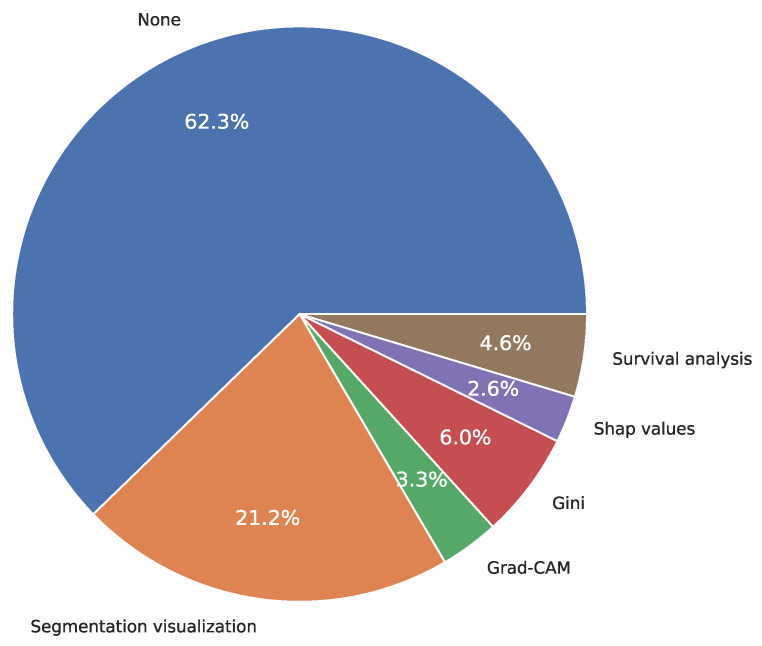
Distribution of explainability tools used in the literature.

Figure 8 shows the several types of explainability tools employed in the reviewed studies. We found that the visualization of image segmentation was commonly used to explain the output of the models (21.2%), followed by Gini coefficients (6.0%), survival analysis (4.6%), Gradient-weighted Class Activation Mapping Grad-CAM (3.3%), and SHAP values with 2.6%. For instance, Ming et al. [139] showed the visualization of CC detection between the prediction result and ground truth under different modal images. In contrast, Mehmood et al. [45] described the most important features associated with CC detection using Gini coefficients. Similarly, Matsuo et al. [98] listed the most important covariates based on the statistical significance associated with CC prediction, whereas Kurita et al. [122] presented a DL-based algorithm for classifying cervical cytological screening; using a Grad-CAM approach allowed visualization of the image components that contributed most to the classification. He et al. [164] utilized the SHAP values approach for the explainability analysis of their predictive model to rank variables in the prediction of cervical precancerous lesions. Interestingly, more than half (62.3%) do not use any explainability approach, which means that these works only presented probabilities without using any tool to open the “black-box” (e.g., [38,46]).

### 3.12. Databases

**Figure 9:**
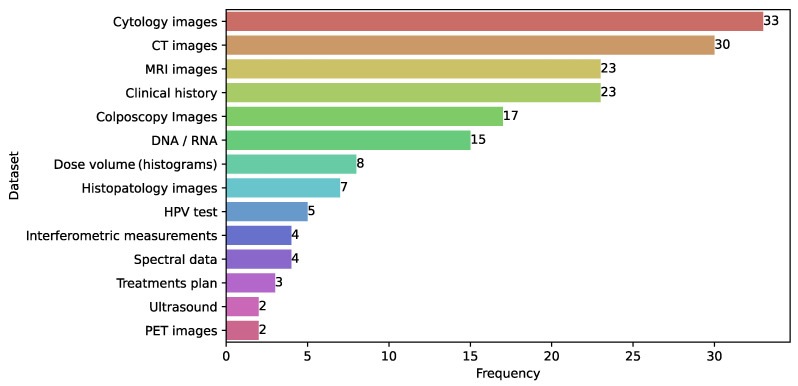
Distribution of the most common databases found in the literature.

Figure 9 highlights the databases used in the development of ML and DL models in CC. Cytology images were the most frequently employed, appearing in 33 studies [39,41], followed by CT images in 30 studies [153,156], MRI images [32,55] and clinical history [43,45] in 23 studies each, and colposcopy images [44,162] in 17 studies.

In particular, the cytology dataset consisted of a set of images to examine abnormal cells that could indicate pre-cancerous changes or cancer [50,63,143]. The CT dataset included images to stratify advanced disease by evaluating lymph nodes [68,156]. For MRI, we found a set of images which were used to evaluate the tumor size, local invasion, and lymph node studies each, as well as colposcopy image involvement [172,182,183]. Related to clinical history, this group can include demographic details, lifestyle, historical medical records, risk factors, and family history of cancer [43,100,164]. Colposcopy images were used to predict and classify cervical lesions [53,70,162]. In the case of DNA/RNA datasets, they included genome sequences, genotyping of cytokines, and T-cell Receptor Sequencing [54,56,62]. Dose volume data refer to the clinical dose administrated to patients [95,124,144]. In histopathology, the dataset consisted of a set of whole-slide biopsy images used to predict lymph node metastasis [118,135].

The use of spectral and genomic data remains less explored in CC predictive modeling. However, spectral data has been primarily employed for diagnosis, while genomic data has been more frequently used for prognosis. Although research predominantly focused on diagnostic applications, images remained the most frequently used input data across all three medical applications. Specifically, 68.24% of diagnostic studies (58/85) used images, 45.71% of prognostic models (16/35) incorporated image-based data, and 70.58% of treatment models (24/34) utilized image inputs.

### 3.13. Limitations

**Figure 10:**
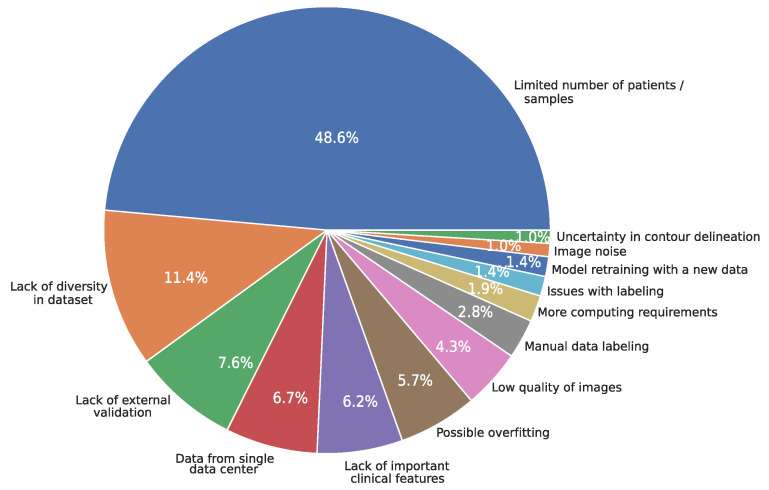
Most common limitations identified in the studies.

Figure 10 summarizes the main limitations found in the reviewed literature. In particular, the limitation most commonly found was the limited number of patients/samples (48.6%) [38,182], followed by a lack of diversity in the dataset (11.4%) [144,156], lack of external validation (7.6%) [132,154], and data from a single data center (6.7%) [164,184]. The least common limitations were image noise [52,142] and uncertainty in contour delineation, each identified in 1% of all studies [59,87].

### 3.14. Reproducibility

Of the 153 studies reviewed, we found that only 6 studies (3.92%) have their programming codes available, compared to 145 studies (94.77%) whose codes are not available. For instance, Wentzensen et al. [66], Jeong et al. [150], and Cheng et al. [51] used a free platform to store and share the code of their DL-based models. In particular, these authors shared the steps to replicate their programming environment, the prerequisites list, requirements (hardware and software), implemented model code, and steps for training, evaluation, and inference. The code available in these studies promotes their reproducibility and can accelerate the research of other scientists.

**Figure 11:**
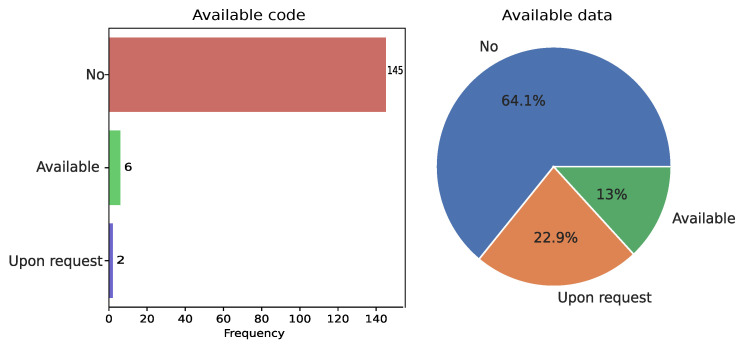
Distribution of available code and dataset.

We also analyzed the distribution of available data and found that only 20 studies (13%) mentioned that their data used during training and testing are freely available. In contrast, 35 studies (22.9%) stated that their data is available upon request, and 98 studies (64.1%) indicated their data is not available. In particular, the reason those studies mention that their data is available is because they used public databases, such as SIPaKMeD [41], Herlev [22,52,82], and Cytology Image Challenge [50], which are Pap Smear datasets. Figure 11 shows the distribution of studies with available code and data.

### 3.15. Distribution of Publications by Country

**Figure 12:**
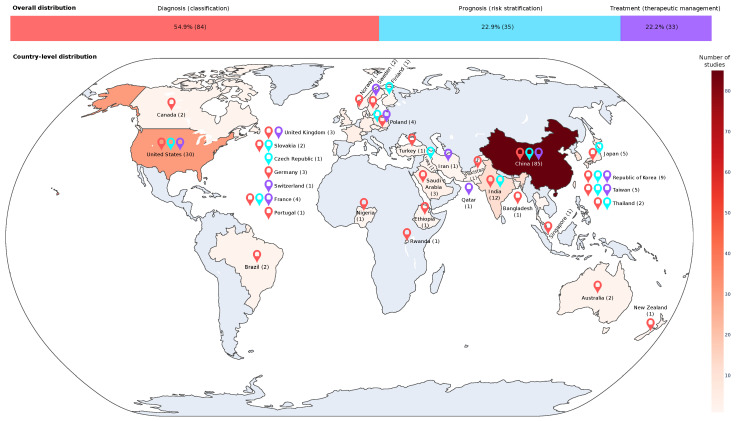
Applications of ML and DL in cervical cancer prediction across countries and targets.

In Figure 12, the distribution of publications by country, research origin, and medical purpose is presented. China had the highest number of studies, totaling 85 (40 focused on diagnosis, 21 on prognosis, and 24 on treatment), followed by the United States with 30 (16 for diagnosis, 4 for prognosis, and 10 for treatment); India conducted 12 studies (10 on diagnosis and 2 on prognosis), while Republic of Korea carried out 9 studies (6 on diagnosis, 2 on prognosis, and 1 on treatment). We observed that countries such as Brazil, Germany, Portugal, Australia, Canada, Nigeria, Norway, Turkey, Saudi Arabia, Ethiopia, Rwanda, Pakistan, Bangladesh, Singapore, and New Zealand presented proposals that addressed only the diagnosis of CC. In contrast, proposals from Czech Republic, Finland, and Iraq addressed only the prognosis of CC. Finally, Sweden, Iran, and Qatar presented studies only for treatments.

Regarding nationality and predictive modeling objectives, only studies involving China, the United States, and France developed models for all three medical applications (diagnosis, prognosis, and treatment). Populations from Japan, Republic of Korea, India, and Thailand had models for diagnostic and prognostic purposes [36,42,56,178], while studies on the Taiwanese population focused on prognosis [88] and treatment [85]. Other populations were exclusively studied for the diagnosis of CC [82,99,148].

### 3.16. Study Design Characteristics

**Figure 13:**
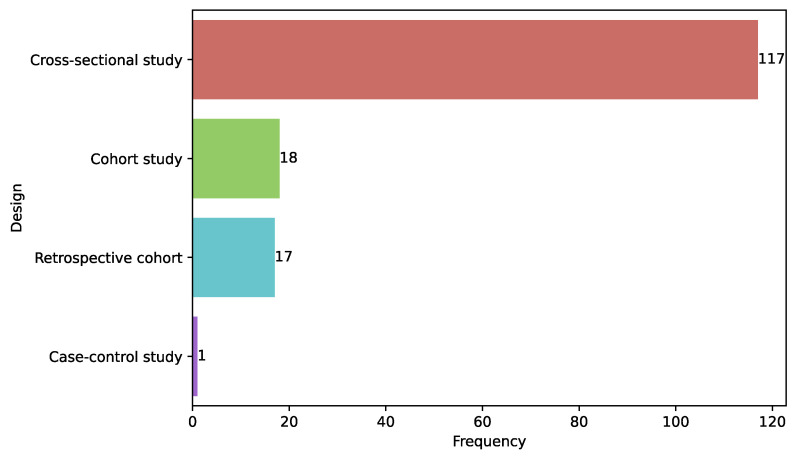
Most common study designs identified in the literature.

Regarding the characteristics of the reviewed studies, all of them were observational in nature. As summarized in Figure 13, the most common study design was cross-sectional (76.47%), followed by cohort studies (11.76%), retrospective cohort studies (11.11%), and a single case–control study (0.65%).

The majority of cross-sectional studies were used to develop diagnostic models for CC (66.67%), followed by models related to treatment (22.22%), and prognostic models (11.11%). For cohort and retrospective cohort studies, the most frequent medical application was prognostic (modeling predicting cancer progression to lymph node metastasis [77,100,136], recurrence [165,169,180], survival [55,58,98], or treatment response [113,175,184]), representing 50% and 88.24%, respectively. This is related to the longitudinal nature of medical care and disease progression. These were followed by treatment-related models (dosage, therapeutic planning, toxicity), with 16.67% for cohort studies and 11.76% for retrospective cohort studies. Finally, 33% of cohort studies were focused on diagnosis, and the only case–control study was developed to predict diagnosis.

### 3.17. Study Population

**Figure 14:**
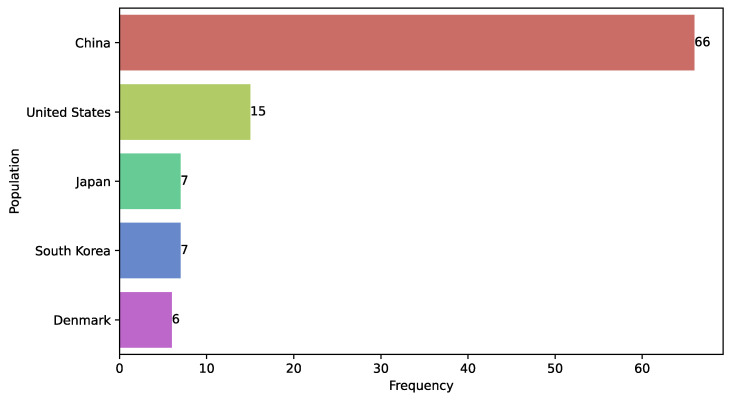
Most frequently studied populations.

A total of 14.38% of the reviewed studies did not report or provide information on the nationality and ethnicity of the study population, while 56.86% did not report the age of recruited patients [58,60,93,94]. We identified that the study populations in the reviewed works originated from 24 different nationalities. Notably, the Chinese population was the most studied, representing 43.14% of the analyses, followed by populations from the United States, Japan, Republic of Korea, and Denmark (Figure 14). The frequency with which some populations were studied is related to the availability of open access datasets. Some studies even used multiple open access databases containing data from populations of different nationalities (e.g., studies [52,82,151]). A relevant example is the Herlev database, which consists of cytological images from Danish patients collected by Herlev University Hospital, leading to limited data diversity across studies.

### 3.18. Medical Specialist Involvement in Predictive Model Development

**Figure 15:**
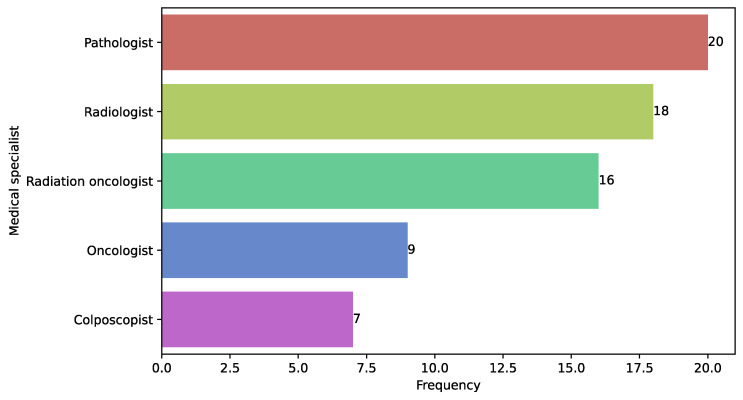
Most frequently involved medical specialists in studies.

To assess the level of medical expert involvement, we explored their participation in study design, implementation, and evaluation. In 40.52% of the studies, there was no explicit reference to the participation of medical specialists. Some studies relied on secondary sources, classifying data based on medical records and notes (e.g., studies [48,91]). However, other studies using secondary data explicitly mentioned specialist involvement for validation or relabeling of patient-sample classifications. Examples include studies by Sornapudi et al. [82] and Shanthi [22], which reclassified cytological images with the assistance of a cytotechnologist and pathologist, respectively. Figure 15 highlights the most frequently referenced profiles across the reviewed studies. In particular, this figure provides a summary of the five most frequently involved medical specialties or specialists, categorizing their participation in different study phases, such as study design (patient recruitment and follow-up), label assignment (cancer staging, delineation, segmentation of key regions), and evaluation (prediction comparison). Some studies involved multiple specialists, such as two pathologists with different levels of experience, validating classification in the same study, or multiple medical profiles [65,83,86], such as an oncologist and radiologist, each performing different tasks within the same study [74,82,84].

## 4. Discussion

**Figure 16:**
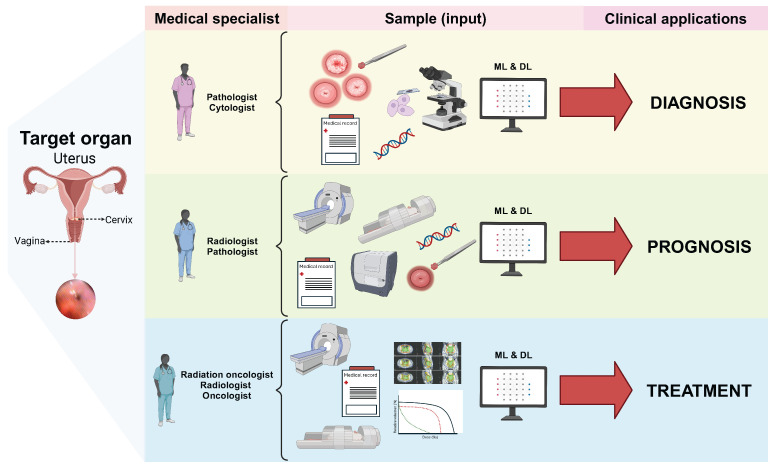
Overview of specialists, inputs, and outputs detected in the reviewed studies by clinical application. Created with BioRender.com.

This scoping review identified that slightly more than half of the studies addressed the application of ML and DL in CC for diagnostic purposes, followed by prognosis and an incipient focus on CC treatment (Figure 16). Within the levels of cancer prevention, it is striking that AI in CC has been aimed at covering needs at the levels of secondary prevention (screening and timely diagnosis) and tertiary prevention (treatment); no study was found with an application approach at the level of primary prevention (HPV vaccines). Less than 20% corresponded to cohort or case–control studies, and another notable finding is that no studies were found that were carried out in Latin America or with a Latino population. To date, few reviews have analyzed the application of ML and DL in CC.

William et al. presented a synthesis of 30 studies up to 2018 that focused on automated detection and classification of CC from pap smear images [185]. A high accuracy of over 90% was reported for existing models, especially for classification as normal or abnormal. However, low accuracy was reported for the classification of some cell classes that cannot be easily identified as normal or abnormal.

Since the manual analysis of pap smear images and microscopic biopsies by a trained cytologist is time-consuming and tedious, as cytotechnologists typically examine a large amount of data daily, the application of ML and DL for CC diagnosis has focused on optimizing the accuracy of CC detection and classification [185]. Similarly, DL models have been developed for dual staining of p16 and Ki-67 (markers that are closely related to cervical carcinogenesis) instead of cytological image identification, considering that the increase in the number of dual-stained cells correlates with increased severity of histopathology, with equal sensitivity and higher specificity compared to cytology and manual evaluation of double-stained slides and a reduction in referrals to colposcopy [66].

Another review published in 2022 focused on presenting the various ML models that had been used for CC prediction until November 2020. In this review, RF, DT, adaptive boosting, and gradient boosting models were reported to have 100% classification scores for CC prediction and 99% accuracy with SVM [186]. Another use of DL has been the segmentation of CC CT images. Radiation therapy is an effective way to improve the survival rate of patients with CC [67,187], especially for patients with locally advanced CC and those whose physical condition is not suitable for surgery. A systematic review and meta-analysis published in 2022 reported good accuracy in the automatic segmentation of CT images in CC with lower time consumption [188].

Manual segmentation of the CTV by a physician is still the standard, but it is a time-consuming and intensive task, taking an experienced physician at least 30 min [188]. Automatic segmentation has shown great potential in reducing physician burden, decreasing patient waiting time, and improving cancer treatment. However, for its use in future radiotherapy applications, high-quality public databases and large-scale research verification are required [188].

On the other hand, another review published in 2023 compiled the synthesis of 13 studies published until October 2022, which evaluated the use of ML to predict survival in patients with CC [189]. The range of the area under the curve reported for overall survival was 0.40–0.99; for disease-free survival, 0.56–0.88; and for progression-free survival, 0.67–0.81. In addition, the combination of heterogeneous multidimensional data with ML techniques demonstrated potential in predicting CC survival. However, despite the benefits of ML, interpretability, explainability, and imbalanced datasets remain some of the biggest challenges. Therefore, for the use of ML models as a standard for survival prediction, further studies are required.

To reduce the risk of mortality from CC, faster diagnosis and accurate prognosis prediction are required. The incorporation of ML and DL models into the health-care system has shown great potential as a support tool in oncology clinical practice [7], and specifically in CC, these AI tools could help in the diagnosis and prediction of the prognosis of the disease as well as shortening the time consumed in the initiation of treatment. Specifically, in Mexico, AI models would be applied within colposcopy clinics where the colposcopic evaluation and biopsy are performed for the diagnosis of precursor lesions, which are the link to obtaining the result of the abnormal screening that will be used to refer patients to oncology clinics to receive treatment. The main challenges, limitations, and future directions identified in the reviewed literature are described below from two perspectives: computational (ML and DL) and clinical challenges.

### 4.1. Computational Challenges of Using ML and DL in CC

In recent years, ML and DL models have made significant progress in medicine and health care, primarily in the development of tools that can assist in clinical decision-making, such as diagnostics of diseases, targeted drug therapy, improvement of progress in clinical trials, and so on [190,191]. This progress has been brought about by three important milestones: (i) the advances in highly parallelizable graphics processing units (GPUs) that enable faster training and increased efficiency of DL models, (ii) development of programming frameworks (e.g., Keras, TensorFlow, PyTorch) that help abstract complex details in neural network implementation by providing libraries available to define network types (e.g., CNN, RNN) and common model architectures, and (iii) advances in DL architectures capable of analyzing multimodal data (e.g., images, text, signals, timeseries) [192,193,194]. Although these milestones mark significant progress, several challenges remain to be addressed to fully realize the potential of ML and DL in the diagnosis, prognosis, and treatment of CC. Table 6 summarizes these limitations, which are described below.

#### 4.1.1. Data Availability

ML approaches, especially DL, typically require large datasets for training in order to learn patterns and achieve high performance [195,196]. A recent review describes DL techniques used in the field of pap smear whole slide imaging (WSI) classification analysis [31]. Most studies use supervised learning to extract features and train their models to identify normal, benign, and malignant cells from medical images. However, the main challenge is that it requires a large amount of labeled data that is not readily available. Unfortunately, many applications in CC have few or limited data to train DL models. As observed in the literature review, the limited number of patients and samples was the main limitation in almost 50% of all studies reviewed. Some strategies to address this challenge include the following: (i) synthetic data generation based on DL models, such as variational autoencoders [197] and generative adversarial network [159], (ii) oversampling for the minority class based on Synthetic Minority Oversampling Technique (SMOTE) [94,167], and (iii) image transformations based on geometric, intensity, and spectral changes.

#### 4.1.2. Data Leakage

Data leakage is a common problem, especially in applications based on ML and DL. This problem is caused by (i) inappropriate feature selection where the selected features are highly correlated with the target, (ii) errors during data preprocessing caused by incorrect data splitting, and (iii) filling missing data with the entire data set [198,199]. One of the main issues of data leakage is inflated performance metrics, such as high accuracy and precision [200]. Some strategies to avoid data leakage in ML and DL studies include [201]: applying data preprocessing to the training and test sets separately (e.g., scale, missing data), properly splitting the training and test sets, ensuring that data from the training set does not appear in the validation or test set, and using cross-validation.

Cross-validation is a technique for evaluating ML and DL models that is crucial to evaluate model performance and prevent overfitting. Overfitting occurs when a model overfits the training data, causing that model to perform extremely well on the data used for training but poorly on new, unknown data [202]. Splitting data into training, validation, and test sets helps to prevent overfitting, to tune hyperparameters, to select the best selection, and to improve the model generalization. Future research should adopt best practices for data splitting to avoid over-optimistic results, mainly in the clinical area.

#### 4.1.3. Limited External Validation

External validation involves assessing whether the predicted model can be adopted in clinical practice [203]. According to Santos et al. [204], models should be evaluated on datasets separate and independent from the data on which the models were trained to increase confidence in their predictions and assess their clinical utility. In the literature review, we found that only 20 (13.07%) studies externally validated their models, meaning that these models may not be generalizable or may not perform well in populations other than those used in training. As noted, the lack of external validation is a major limitation of the reviewed studies; therefore, future research should include external validation to ensure the clinical relevance and generalization of the models.

#### 4.1.4. Limited Evaluation of Model Performance

Performance metrics are important in evaluating the success or failure of developed models using ML and DL [205]. The metrics help to evaluate the predictive power of a developed model as well as to assess the reliability and rigor of a study [206]. In the reviewed studies, the most common metric used was accuracy, in 77 studies; however, in the clinical area, the confusion matrix is crucial for evaluating the performance of classification models, due to identifying the balance between false positives and false negatives [207]. It is important to highlight the importance of confusion matrices in classification tasks, which should be included in future research for both validation and test sets. If possible, confusion matrices for external validation sets should also be included. These matrices allow for detailed analysis of model performance, especially with respect to false positives and false negatives, which are crucial in the clinical setting.

The learning curve is another common metric for evaluating the performance of a machine learning algorithm. This curve assesses the model’s convergence and diagnoses’ overfitting or underfitting during training [208]. Similar to the confusion matrix, the learning curves should be included in future studies to assess the convergence of the model, identify overfitting/underfitting, provide transparency, and allow readers to assess the generalization of the models.

#### 4.1.5. Complex Data

One of the most common challenges in developing ML and DL models in CC is the complexity and high dimensionality of the data. As observed in Figure 9, the data can be represented as images, time series, spectral data, clinical history, laboratory tests, treatment plans, and so on. Each of these data contains valuable information; however, processing each type of data involves performing specific preprocessing tasks. For instance, the image processing can include resizing, normalization, noise reduction, and geometric transformations (for data augmentation) with the objective of improving the quality of the images [49,52,59,60]. In contrast, for clinical history, the processing tasks can involve feature selection, data standardization, missing data, and sometimes natural language processing in free-text notes [46,94,108]. Data in clinical settings are characterized by their high dimensionality and complexity; future research should adopt best practices to address this issue by applying specific preprocessing tasks to develop ML and DL-based solutions.

#### 4.1.6. Privacy Issues

Clinic data contain highly sensitive and confidential information that identifies the patient and their medical condition or treatment [209,210]. However, several studies have pointed out that DL-based models can be subjected to reverse engineering techniques in order to extract sensitive information used during training [211,212]. The development of technologies that integrate cybersecurity tools is necessary to assess these risks and prevent them, ensuring patient privacy.

#### 4.1.7. Lack of Explainability

In recent years, the performance of ML- and DL-based models has improved dramatically; however, they still often suffer from poor explainability, which limits their applicability in critical areas [213]. Explainability refers to the characteristic that ML and DL-based models must present to be interpreted by a human [213]. One of the main issues of DL-based models is that they are “black-box”, which means they are non-intuitive and difficult for people to understand [214]. This challenge generates a barrier to the application of these models in clinical practice due to lack of interpretability, trust, and transparency [214,215]. In this review, we found that 62.3% of the studies do not use any explainability approach to clearly explain the model’s decision-making (Figure 8), which could limit their use in clinical areas. Future work should consider the use of explainability approaches to increase the confidence and applicability of ML and DL-based models in real-world scenarios.

#### 4.1.8. Lack of Reproducibility

According to Beam et al. [216], reproducibility is a prerequisite for the creation of new knowledge and scientific progress. A study is considered reproducible when an independent team, provided with the original data and code, is able to achieve the same findings as those reported in the initial research [216]. In the reviewed literature, we found that few studies share their code and data (Figure 11), which represents a challenge to reproducing these studies. Mislan et al. [217] mentioned that making code and data available allows analyses to be more easily reproduced and can accelerate the research of other scientists. It is important that future research promotes reproducibility and reuse of codes, which could improve the quality and accelerate the pace of CC research.

### 4.2. Clinical Implementation Challenges of Using ML and DL in CC

From the clinical perspective of the specialists who are the authors of this review, the application of ML and DL closest to CC is in diagnosis as a support tool for secondary prevention of the disease in the diagnosis of premalignant lesions. However, studies are required to demonstrate their reproducibility and internal and external validity for their long-term applicability in therapeutic management and for the monitoring and prediction of patients’ prognoses. In summary, the use of ML and DL in oncology care is a turning point in advanced medical care, and their application in CC will have a deep impact on the lives of CC patients when their validity and reproducibility make them available for use in the clinical setting and data can be translated into therapy. This will especially be the case in more remote environments that are less favored in terms of care and face a shortage of personnel: cytologists, pathologists, and colposcopists. Table 7 describes the limitations for clinical implementation.

#### 4.2.1. Representativeness of Clinical Stages of CC in the Training of DL-Based Models

Although molecular HPV detection testing is the recommended primary screening method for CC [218], and in some countries, cytology combined with screening for high-risk HPV subtypes at five-year intervals is recommended to improve the positive predictive value of screening, there are still excessive referrals for colposcopy due to the high prevalence of transient HPV infections [219]. In resource-limited settings, the use of alternative low-cost methodologies such as visual inspection with acetic acid (VIA) is indicated; however, it has low accuracy and reproducibility, so the development of automated visual assessment based on DL of cervical images could help improve the accuracy and reproducibility of VIA as an assistive technology. However, this technology requires some clinical and technical considerations for it to be widely used as a clinical test, including the training of deep learning-based models with representative images of each of the four distinct biological stages of CC: normal cervix, high-risk HPV infection, precancer, and invasive CC [220]. In this regard, Desai et al. described the essential clinical and technical considerations involved in building a DL-based automated visual evaluation (AVE) tool validated for broad use as a clinical test [220]. In particular, it emphasizes the need for a number of representative images with truth labels necessary to build an accurate but generalizable DL-based automated visual assessment algorithm for cervical images using a DL approach.

#### 4.2.2. Privacy Concerns and Data Security in Health Care

As described above, ML and DL are AI applications that can help identify patterns and trends in health data to improve the diagnosis and treatment of CC. However, since a large amount of personal data is used to train AI models, there is a risk to patient privacy and security, as sensitive medical data are collected and stored, increasing the risk of cyberattacks and information breaches. Therefore, the guidelines for the responsible use of AI in public health developed by the World Health Organization (WHO) and FDA should be considered [221]. These guidelines include the need for a careful assessment of the risks and benefits of AI in health care, transparency in the development and use of AI, and the need to ensure equity and nondiscrimination in health care.

#### 4.2.3. Integration of AI with Clinical Workflows for Real-Time Decision-Making

According to the WHO, AI-assisted cervical cancer screening improves techniques involving the visual evaluation of digital images [222]. This is particularly relevant in low-resource areas with a shortage of specialized personnel. However, the challenge lies in access to computing resources and services for implementing ML and DL models on mobile devices or laptops in remote areas and in the willingness of health care professionals to adopt AI and make real-time clinical decisions when analyzing a specific case [223].

#### 4.2.4. Ethical and Regulatory Considerations

Among the key ethical considerations in the clinical implementation of digital technologies for CC screening using ML and DL are safety monitoring, data privacy, traceability, accountability, and patient protection. These considerations are essential to uphold the principles of beneficence, nonmaleficence, autonomy, and justice [224]. Respecting patient autonomy supports the safeguarding of privacy, the maintenance of confidentiality, and the assurance of informed and valid consent to protect individuals’ data. AI technologies should also be as explainable as possible and tailored to the level of understanding of their intended users. Moreover, these systems must undergo continuous, systematic, and transparent evaluation to ensure their effectiveness and appropriateness in the specific contexts in which they are deployed. From a regulatory perspective, ML and DL models often face challenges, such as limited generalizability beyond their training data and vulnerability to bias. Therefore, health care institutions must develop comprehensive strategies for AI implementation that address the management of technological infrastructure, cost considerations, and the integration of AI systems into existing clinical workflows [224].

#### 4.2.5. Public Perspectives on Using AI in Diagnoses Decisions

Considering that the primary application of ML and DL in CC is in screening, and that current AI models achieve an estimated classification accuracy of 85% for cervical lesions, several critical needs must be addressed. These include ensuring algorithm accountability, improving model performance to increase specificity, and enhancing the accurate classification of premalignant cervical lesions to reduce the risk of misdiagnosis. Furthermore, robust validation of AI systems is essential to demonstrate their effectiveness at the population level [225].

### 4.3. Limitations

Our scoping review has some limitations. This scoping review is a qualitative review of the literature reported up to April 2025. Furthermore, this study focused only on ML and DL approaches. We conducted a search in four selected databases (IEEE, Web of Sciences, Scopus, PubMEd).

## 5. Conclusions

This scoping review gathered relevant scientific evidence on ML and DL applications in CC. AI in the prevention, diagnosis, and treatment process of CC would support timely medical care in hard-to-reach areas with less human capital and help doctors and the health system to reduce administrative burden, care time, professional burnout, and human error. It would also improve the correlation of histological study and the precision of diagnosis and therapeutic management. From 153 reviewed studies, we presented the main challenges and future works from two perspectives: computational and clinical challenges. From the computational view, we identified eight challenges: data availability, data leakage, limited external validation, limited evaluation of model performance, complex data, privacy issues, lack of explainability, and lack of reproducibility. From the clinical view, we found five challenges: representative of clinical stages of CC, privacy concerns, integration of IA with clinical workflows, ethical considerations, and public perspective.

Although ML and DL’s potential use in the diagnosis, prognosis, and treatment of CC has been reported with a significant future impact on oncology clinical practice that allows data to be translated into therapy, more evidence of validity and reproducibility is required for their use in the early detection, prognosis, and therapeutic management of CC. It is important to emphasize that the implementation of AI in public health must be careful and ethical, ensuring that patients’ rights and privacy are respected.

## Figures and Tables

**Table 1:** The quality assessment form used in the scoping review for observational studies assigned scores as follows for questions 1, 2, 4, 6, 7, 8, 9, 10, and 11: 0, no description; 1, limited description; and 2, comprehensive description.

Section	Question
Introduction	**Q1** Is the scientific background adequately described? **Q2** Are the goals clearly outlined?
Methods	**Q3** What is the study design? (1 cross-sectional; 2 case–control; 3 cohort study; 4 clinical trial) **Q4** Are the inclusion criteria and participant selection clearly outlined? **Q5** Sample size (0 if <20, 1 if between 20 and 100, 2 if >100) **Q6** Is the method (validity) explained? **Q7** Are the statistical analyses suitable?
Results	**Q8** Are subjects’ characteristics provided? **Q9** Are the results understandable?
Discussion	**Q10** Are the study results compared and discussed in relation to other studies published in the literature? **Q11** Are study limitations discussed?

**Table 2:** Quality assessment analysis using STROBE scale for observational studies.

Ref.	Q1	Q2	Q3	Q4	Q5	Q6	Q7	Q8	Q9	Q10	Q11	Total
Kim et al. 2022 [36]	2	2	1	1	2	2	2	2	2	1	0	17
Yuan et al. 2022 [37]	1	2	1	1	1	0	1	1	1	1	0	10
Kruczkowski et al. 2022 [38]	2	2	1	2	2	1	0	1	1	1	0	13
Yoganathan et al [32]	1	2	1	1	1	2	0	2	2	2	0	14
Fu et al. 2022 [39]	1	2	2	1	2	1	2	2	2	1	0	16
Ma et al. 2022 [40]	2	2	1	2	2	1	2	2	2	2	2	20
Liu et al. 2022 [41]	1	2	1	1	2	0	1	1	1	1	0	11
Nambu et al. 2022 [42]	2	2	1	1	2	1	2	1	2	2	2	18
Drokow et al. 2022 [43]	1	2	1	1	2	0	1	1	1	1	0	11
Liu et al. 2022 [44]	2	2	1	1	2	1	1	2	2	1	2	17
Mehmood et al. 2021 [45]	2	2	1	0	2	1	1	0	2	1	0	12
Ali et al. 2021 [46]	1	2	1	1	2	0	1	1	1	1	0	11
Chu et al. 2021 [47]	2	2	3	2	2	2	2	2	2	2	2	23
Dong et al. 2021 [48]	1	2	1	1	2	0	1	1	1	1	0	11
Fick et al. 2021 [49]	1	2	1	1	2	0	1	1	1	1	0	11
Chen et al. 2021 [50]	2	2	1	1	1	0	1	1	2	2	0	13
Cheng et al. 2021 [51]	2	2	1	1	2	1	1	1	2	2	0	15
Rahaman et al. 2021 [52]	2	2	1	0	2	1	1	1	1	1	2	14
Park et al. 2021 [53]	2	2	1	1	2	1	2	2	2	2	2	19
Tian et al. 2021 [54]	2	2	1	0	2	1	0	0	1	1	0	10
Da-ano et al. 2021 [55]	2	2	3	1	2	1	1	2	1	1	2	18
Kaushik et al. 2021 [56]	2	2	1	0	2	1	1	1	1	1	0	12
Shi et al. 2021 [57]	2	2	3	1	2	1	1	1	1	1	2	17
Ding et al. 2021 [58]	2	2	1	0	2	1	1	1	1	1	0	12
Zhu et al. 2021 [59]	2	2	1	0	2	1	1	1	1	1	0	12
Chandran et al. 2021 [60]	2	2	1	0	2	1	2	1	1	1	2	15
Jian et al. 2021 [61]	2	2	1	0	2	1	1	1	1	1	1	13
Christley et al. 2021 [62]	2	2	3	1	2	1	2	1	1	1	1	17
Ke et al. 2021 [63]	2	2	1	1	2	1	1	1	1	1	2	15
Rigaud et al. 2021 [64]	2	2	1	1	3	1	1	1	1	1	2	16
Urushibara et al. 2021 [65]	2	2	1	1	2	1	1	1	1	1	2	15
Wentzensen et al. 2021 [66]	2	2	1	1	2	1	1	1	1	1	2	15
Wang et al. 2020 [67]	2	2	1	1	2	1	1	1	1	1	2	15
Liu et al. 2020 [68]	2	2	1	1	2	1	1	1	1	1	1	14
Mao et al. 2020 [69]	2	2	1	1	1	1	1	1	1	1	1	13
Xue et al. 2020 [70]	2	2	1	1	2	1	1	1	1	1	2	15
Bao et al. 2020 [71]	2	2	1	1	2	1	1	1	1	1	2	15
Cho et al. 2020 [72]	2	2	1	1	2	1	1	1	1	1	2	15
Ju et al. 2020 [73]	2	2	1	1	2	1	1	1	1	1	1	14
Kanai et al. 2020 [74]	2	2	1	1	1	1	1	1	1	1	1	13
Yuan et al. 2020 [75]	2	2	1	1	2	1	1	1	1	1	1	14
Hu et al. 2020 [76]	2	2	1	1	2	1	1	1	1	1	1	14
Wu et al. 2020 [77]	2	2	3	1	2	1	1	1	1	1	2	17
Wang et al. 2020 [78]	2	2	3	1	2	1	1	1	1	1	1	16
Kudva et al. 2020 [79]	2	2	1	1	2	1	1	1	1	1	1	14
Ijaz et al. 2020 [80]	2	2	1	1	2	1	1	1	1	1	1	14
Bae et al. 2020 [81]	2	2	1	1	2	1	1	1	1	1	1	14
Sornapudi et al. 2020 [82]	2	2	1	1	2	1	1	1	1	1	1	14
Shao et al. 2020 [83]	2	2	1	1	1	1	1	1	1	1	1	13
Takada et al. 2020 [84]	2	2	3	1	1	1	1	1	1	1	2	16
Lin et al. 2020 [85]	2	2	1	1	2	1	1	1	1	1	1	14
Jihong et al- 2020 [86]	2	2	1	1	2	1	1	1	1	1	1	14
Liu et al. 2020 [87]	2	2	1	1	2	1	1	1	1	1	1	14
Shen et al. 2019 [88]	2	2	3	1	2	1	1	1	1	1	2	17
Pathania et al. 2019 [89]	2	2	1	1	2	1	1	1	1	1	2	15
Aljakouch et al. 2019 [90]	2	2	1	1	1	1	1	1	1	1	1	13
Shanthi et al. 2019 [22]	2	2	1	1	2	1	1	1	1	1	1	14
Dong et al. 2019 [91]	2	2	1	1	2	1	1	1	1	1	1	14
Hu et al. 2019 [92]	2	2	3	1	2	1	1	1	1	1	2	17
Dong et al. 2019 [93]	2	2	1	1	2	1	1	1	1	1	1	14
Geetha et al. 2019 [94]	2	2	1	1	2	1	1	1	1	1	1	14
Shen et al. 2019 [95]	2	2	1	1	1	1	1	1	1	1	1	13
Zhang et al. 2019 [96]	2	2	1	1	2	1	1	1	1	1	1	14
Chen et al. 2019 [97]	2	2	1	1	1	1	1	1	1	1	1	13
Matsuo et al. 2019 [98]	2	2	3	1	2	1	1	1	1	1	1	16
Araujo et al. 2019 [99]	2	2	1	1	2	1	1	1	1	1	1	14
Kan et al. 2019 [100]	2	2	3	1	2	1	1	1	1	1	1	16
Zhen et al. 2017 [101]	2	2	3	1	2	1	1	1	1	1	1	16
Xie et al. 2018[102]	2	2	1	1	1	1	1	1	1	1	1	13
Kudva et al. 2018 [103]	2	2	1	1	2	1	1	1	1	1	1	14
Wei et al. 2017 [104]	2	2	1	1	1	1	1	1	1	1	1	13
Guo et al. 2016 [105]	2	2	1	1	1	1	1	1	1	1	1	13
Zhao et al. 2016 [106]	2	2	1	1	2	1	1	1	1	1	1	14
Weegar et al. 2015 [107]	2	2	3	1	2	1	1	1	1	1	1	16
Kahng et al. 2015 [108]	2	2	3	1	2	1	1	1	1	1	1	16
Mu et al. 2015 [109]	1	2	1	1	1	1	1	1	1	1	1	12
Zhi et al. 2015 [110]	2	2	1	1	2	1	1	1	1	1	1	14
Dong et al. 2015 [111]	2	2	1	1	2	1	1	1	1	1	1	14
Mariarputham et al. 2015 [112]	2	2	1	1	2	1	1	1	1	1	1	14
Xin et al. 2023 [113]	2	2	4	1	2	1	1	1	1	1	1	17
Robinson et al. 2023 [114]	2	2	1	1	1	1	1	1	1	1	1	13
Tian et al. 2023 [115]	2	2	1	1	2	1	1	1	1	1	1	14
Kaur et al. 2023 [116]	2	2	1	1	2	1	1	1	1	1	1	14
Ji et al. 2023 [117]	2	2	1	1	2	1	1	1	1	1	1	14
Liu et al. 2023 [118]	2	2	3	1	2	1	1	1	1	1	2	17
Kang et al. 2023 [119]	2	2	1	1	2	1	1	1	1	1	1	14
Wu et al. 2023 [120]	2	2	1	1	2	1	1	1	1	1	1	14
Ince et al. 2023 [121]	2	2	1	1	1	1	1	1	1	1	1	13
Kurita et al. 2023 [122]	2	2	1	1	2	1	1	1	1	1	1	14
Zhu et al. 2023 [123]	2	2	3	1	2	1	1	1	1	1	2	17
Yang et al. 2023 [124]	2	2	1	1	1	1	1	1	1	1	1	13
Devi et al. 2023 [125]	2	2	1	1	2	1	1	1	1	1	1	14
Chen et al. 2023 [126]	2	2	3	1	2	1	1	1	1	1	2	17
Salehi et al. 2023 [127]	2	2	1	1	1	1	1	1	1	1	1	13
Yu et al. 2023 [128]	2	2	3	1	2	1	1	1	1	1	1	16
Liu et al. 2023 [129]	2	2	4	1	2	1	1	1	1	1	1	17
Wang et al. 2023 [130]	2	2	3	1	2	1	1	1	1	1	2	17
Yu et al. 2023 [131]	2	2	1	1	2	1	1	1	1	1	2	15
Huang et al. 2023 [132]	2	2	1	1	1	1	1	1	1	1	1	13
Cao et al. 2023 [133]	2	2	1	1	2	1	1	1	1	1	1	14
Chen et al. 2022 [134]	2	2	3	1	2	1	1	1	1	1	2	17
He et al. 2023 [135]	2	2	1	1	1	1	1	1	1	1	1	13
Qian et al. 2022 [136]	2	2	3	1	2	1	1	1	1	1	2	17
Dong et al. 2022 [137]	2	2	3	1	2	1	1	1	1	1	2	17
Ji et al. 2022 [138]	2	2	1	1	1	1	1	1	1	1	1	13
Ming et al. 2022 [139]	2	2	1	1	2	1	1	1	1	1	1	14
Wang et al. 2022 [140]	2	2	1	1	2	1	1	1	1	1	2	15
Ma et al. 2022 [141]	2	2	1	1	2	1	1	1	1	1	1	14
Chang et al. 2022 [142]	2	2	1	1	2	1	1	1	1	1	1	14
Tao et al. 2022 [143]	2	2	1	1	2	1	1	1	1	1	1	14
Qilin et al. 2022 [144]	2	2	1	1	2	1	1	1	1	1	1	14
Xue et al. 2023 [145]	2	2	1	1	1	1	1	1	1	1	1	13
Gay et al. 2023 [146]	2	2	1	1	2	1	1	1	1	1	1	14
Shen et al. 2024 [147]	2	2	1	1	2	1	1	1	1	1	1	14
Aljrees et al. 2024 [148]	2	2	1	1	1	1	1	1	1	1	1	13
Munshi et al. 2024 [149]	2	2	1	1	1	1	1	1	1	1	1	13
Jeong et al. 2024 [150]	2	2	1	1	1	1	1	1	1	1	1	13
Stegmuller et al. 2023 [151]	2	2	1	1	1	1	1	1	1	1	1	13
Wu et al. 2024 [152]	2	2	3	1	2	1	1	1	1	1	2	17
Wu et al. 2025 [153]	2	2	4	1	2	1	1	1	1	1	2	18
Xin et al. 2024 [154]	2	2	3	1	2	1	1	1	1	1	2	17
Chen et al. 2018 [155]	2	2	1	1	1	1	1	1	1	1	2	14
Chen et al. 2020 [156]	2	2	1	1	2	1	1	1	1	1	2	15
Matsuo et al. 2019 [98]	2	2	3	1	2	1	1	1	1	1	2	17
Zheng et al. 2024 [157]	2	2	1	1	2	1	1	1	1	1	2	15
Brenes et al. 2024 [158]	2	2	1	1	2	1	1	1	1	1	2	15
Shandilya et al. 2024 [159]	2	2	1	1	2	1	1	1	1	1	2	15
Mathivanan et al. 2024 [160]	2	2	1	1	2	1	1	1	1	1	2	15
Wang et al. 2021 [161]	2	2	3	1	2	1	1	1	1	1	2	17
Dong et al. 2025 [162]	2	2	1	1	2	1	1	1	1	1	2	15
Xiao et al. 2023 [163]	2	2	1	1	2	1	1	1	1	1	2	15
He et al. 2024 [164]	2	2	3	1	2	1	1	1	1	1	2	17
Wang et al. 2024 [165]	2	2	3	1	2	1	1	1	1	1	2	17
Wu et al. 2021 [166]	2	2	3	1	2	1	1	1	1	1	2	17
Namalinzi et al. 2024 [167]	2	2	1	1	2	1	1	1	1	1	2	15
Liu et al. 2023 [168]	2	2	1	1	2	1	1	1	1	1	2	15
Senthilkumar et al. 2021 [169]	2	2	1	1	2	1	1	1	1	1	2	15
Suvanasuthi et al. 2025 [170]	2	2	1	1	1	1	1	1	1	1	2	14
Du et al. 2020 [171]	2	2	1	1	2	1	1	1	1	1	2	15
Liu et al. 2024 [172]	2	2	1	1	2	1	1	1	1	1	2	15
Yi et al. 2022 [173]	2	2	1	1	2	1	1	1	1	1	2	15
Ye et al. 2024 [174]	2	2	1	1	2	1	1	1	1	1	2	15
Guo et al. 2023 [175]	2	2	1	1	2	1	1	1	1	1	2	15
Hang et al. 2021 [176]	2	2	3	1	1	1	1	1	1	1	2	16
Ramesh et al. 2022 [177]	2	2	1	1	2	1	1	1	1	1	2	15
Monthatip et al. 2023 [178]	2	2	1	1	2	1	1	1	1	1	2	15
Felix et al. 2024 [179]	2	2	1	1	1	1	1	1	1	1	2	14
Kawahara et al. 2022 [180]	2	2	3	1	1	1	1	1	1	1	2	16
Zhang et al. 2024 [181]	2	2	1	1	1	1	1	1	1	1	2	14
Park et al. 2020 [182]	2	2	3	1	1	1	1	1	1	1	2	16
Kruczkowski et al. 2022 [38]	2	2	1	1	1	1	1	1	1	1	2	14
Zhang et al. 2024 [183]	2	2	1	1	1	1	1	1	1	1	2	14
Cai et al. 2024 [184]	2	2	3	1	2	1	1	1	1	1	2	17

**Table 3:** Summary of ML-based applications reported in the literature.

Ref.	Year	Clinical Application	Prediction Task	Target	Datasets	No. Folds for CV	Best Performance on Test Set		Extval
							**Model**	**Metric**	
[170]	2025	Diagnosis	Classification	Screening for CC	DNA	10	RF	ACC = 90.9%	-
[148]	2024	Diagnosis	Classification	Screening for CC	Clinical history	5	KNN	ACC = 99%	-
[149]	2024	Diagnosis	Classification	Screening for CC	Clinical history	5	SVM	ACC = 99%	-
[154]	2024	Prognosis	Classification	Survival	MRI images	-	RF	ACC = 86%	-
[164]	2024	Prognosis	Classification	Cancer progression	Clinical history	10	RF	ACC = 86%	-
[165]	2024	Treatment	Classification	Recurrence (Cancer progression)	DNA	10	RF	ACC = 84%	-
[167]	2024	Diagnosis	Classification	Screening for CC	Clinical history	-	RF	ACC = 90%	-
[172]	2024	Prognosis	Classification	Screening for CC	MRI images	10	SVM	AUC = 76%	Yes
[174]	2024	Treatment	Classification	Therapeutic dose and planning	DNA	10	RF	ACC = 96%	-
[179]	2024	Diagnosis	Classification	Stages of CC	Dose volume	5	SVM	ACC = 96%	-
[181]	2024	Prognosis	Classification	Stages of CC	DNA	-	Lightgbm	AUC = 98.7%	-
[113]	2023	Prognosis	Classification	Cancer progression	MRI images	5	SVM	ACC = 90%	Yes
[121]	2023	Diagnosis	Classification	Stages of CC	MRI images	5	SVM	ACC > 83%	-
[123]	2023	Diagnosis	Classification	Stages of CC	Spectral data	10	SVM	ACC = 95%	-
[124]	2023	Treatment	Classification	Therapeutic dose and planning	CT images	-	AAA	ACC = 73%	-
[125]	2023	Diagnosis	Classification	Stages of CC	Clinical history	5	LR DT	ACC > 88%	-
[128]	2023	Prognosis	Regression	Survival	Histopatology images Clinical history	5	XGBoost	AUC = 83%	-
[135]	2023	Diagnosis	Classification	Stages of CC	Histopathology images	-	SVM	ACC > 87%	-
[168]	2023	Prognosis	Classification	Cancer progression	MRI images Clinical history	5	MNB	ACC = 77%	-
[175]	2023	Treatment	Classification	Cancer progression	DNA	-	RF	-	-
[178]	2023	Prognosis	Classification	Cancer progression	CT images Clinical history	10	SVM	ACC = 90.1%	-
[38]	2022	Diagnosis	Classification	Stages of CC	Interferometry	3	NB	ACC = 92%	-
[39]	2022	Diagnosis	Classification	Screening for CC	Colposcopy images Cytology images HPV test	-	MLR	AUC = 92.1%	-
[138]	2022	Diagnosis	Classification	Stages of CC	Fluorescence images	-	KNN	SEN = 90%	-
[173]	2022	Prognosis	Classification	Cancer progression	Ultrasound	-	SVM	ACC = 85%	-
[177]	2022	Diagnosis	Classification	Stages of CC	DNA	-	SVM	ACC = 91.5%	-
[180]	2022	Prognosis	Classification	Recurrence (Cancer progression)	MRI images	5	LASSO	ACC = 93.1%	-
[38]	2022	Prognosis	Classification	Stages of CC	Dose volume	3	NB	ACC = 92%	-
[46]	2021	Diagnosis	Classification	Screening for CC	Biopsy results Cytology images	-	RT RF KNN	ACC = 95.5%	-
[47]	2021	Prognosis	Classification	Cancer progression	Histopathology images Clinical history	5	LR SVM	AUC = 88%	-
[55]	2021	Prognosis	Classification	Surv forest	MRI images CT images Clinical history	10	RF	AUC > 84%	-
[56]	2021	Prognosis	Classification	Cancer progression	DNA	5	Ridge	ACC = 84.7%	-
[58]	2021	Prognosis	Classification	Survival	DNA	10	SVM	AUC > 91%	-
[62]	2021	Diagnosis	Classification	Cancer progression	DNA	LOOCV	LR	ACC = 95%	-
[166]	2021	Diagnosis	Classification	Screening for CC	Ultrasound	-	LR	AUC = 91%	Yes
[176]	2021	Prognosis	Classification	Recurrence (Cancer progression)	DNA	-	SVM	ACC = 83%	Yes
[74]	2020	Diagnosis	Classification	Stages of CC	Cytology images	10	LSVM	ACC = 84%	-
[78]	2020	Prognosis	Classification	Cancer progression	MRI images	10	SVM	C-index 0.96	-
[80]	2020	Diagnosis	Classification	Screening for CC	Clinical history	10	RF	ACC > 95%	-
[81]	2020	Diagnosis	Classification	Stages of CC	Colposcopy images	10	KNN	ACC = 80%	-
[156]	2020	Prognosis	Classification	Cancer progression	CT iimages	-	SVM	ACC = 76%	-
[171]	2020	Diagnosis	Classification	Screening for CC	DNA	10	DT	SEN = 88.6%	Yes
[182]	2020	Prognosis	Classification	Survival	MRI images	-	SF	AUC = 79.6%	-
[94]	2019	Diagnosis	Classification	Screening for CC	Clinical history	10	RF	ACC > 94%	-
[100]	2019	Prognosis	Classification	Cancer progression	MRI images Clinical history	-	SVM	AUROC = 75%	-
[104]	2017	Diagnosis	Classification	Stages of CC	Histopathology images	-	SVM	ACC > 90%	-
[105]	2016	Diagnosis	Classification	Stages of CC	Histopathology images	10	SVM	ACC = 88.5%	-
[106]	2016	Diagnosis	Classification	Screening for CC	Cytology images	10	SVM	ACC = 98%	-
[108]	2015	Prognosis	Classification	Cancer progression	Clinical history	10	SVM	ACC = 74%	-
[109]	2015	Treatment	Image segmentation	Segmentation of targets/OARs	CT images	LOOCV	SVM	DSC = 91.78	-
[111]	2015	Prognosis	Classification	Cancer progression	DNA	5	SVM	ACC = 80%	-
[112]	2015	Diagnosis	Classification	Stages of CC	Cytology images	10	SVM	Precision >87%	-

**Table 4:** Summary of DL-based applications reported in the literature.

Ref.	Year	Clinical Application	Prediction Task	Target	Datasets	No. Folds For CV	Best Performance on Test Set		Externalval
							**Model**	**Metric**	
[147]	2024	Treatment	Classification	Therapeutic dose and planning	CT images Treatment plan Dose volume	-	U-Net	AUC = 94%	-
[150]	2024	Prognosis	Classification	Cancer progression	MRI images	5	CNN	ACC = 78%	-
[152]	2024	Prognosis	Regression	Cancer progression	CT images	5	DNN	ACC = 75%	-
[153]	2024	Treatment	Image Segmentation	Delineation of the CTV	CT images	-	ResCANet	DSC = 74.8	Yes
[157]	2024	Diagnosis	Classification	Stages of CC	DNA	-	EfficientNet DenseNet InceptionNet	ACC = 94.4%	-
[158]	2024	Diagnosis	Image Segmentation	Screening for CC	Colposcopy images	-	Efficient U-Net LSTM-Attention	ACC = 87%	-
[159]	2024	Diagnosis	Classification	Stages of CC	Cytology images	-	CNN	ACC = 99.11%	-
[183]	2024	Treatment	Classification	Therapeutic dose and planning	MRI images	10	ResNet101 MLP	AUC = 87%	-
[114]	2023	Diagnosis	Image Segmentation	Stages of CC	Interferometric Measurements	LOOCV	CNN	ACC = 81%	-
[115]	2023	Treatment	Image Segmentation	Delineation of the CTV	CT images	5	AFN	DSC > 88	-
[116]	2023	Diagnosis	Classification	Stages of CC	Cytology images	-	MLNet	ACC > 99%	Yes
[117]	2023	Diagnosis	Image Segmentation	Screening for CC	Cytology images	-	CNN	DSC = 0.94	-
[118]	2023	Treatment	Classification	Therapeutic dose and planning	Histopathology images	-	ViT RNN	ACC = 90%	Yes
[119]	2023	Diagnosis	Classification	Stages of CC	Spectral data	5	CNN	ACC = 94%	-
[120]	2023	Diagnosis	Classification	Stages of CC	Spectral data	LOOCV	DBN	ACC = 93%	-
[122]	2023	Diagnosis	Classification	Stages of CC	Cytology images	-	CNN	ACC = 87%	-
[126]	2023	Diagnosis	Classification	Stages of CC	Colposcopy images	-	Efficient Net GRU	ACC = 91%	-
[127]	2023	Treatment	Image Segmentation	Therapeutic dose and planning	CT images	-	CNN	Jaccard > 0.86	-
[129]	2023	Diagnosis	Classification	Screening for CC	Histopatology images	5	CNN	ACC = 76%	-
[130]	2023	Treatment	Image Segmentation	Segmentation of targets/OARs	CT images	-	CNN	DSC = 0.77	Yes
[131]	2023	Treatment	Regression	Therapeutic dose and planning	CT images	-	3DResUnet	Dose = 5%	-
[132]	2023	Diagnosis	Image Segmentation	Segmentation of targets/OARs	CT images	4	CNN	DSC = 0.80	-
[133]	2023	Diagnosis	Classification	Stages of CC	Colposcopy images	-	CNN	ACC > 88%	-
[145]	2023	Diagnosis	Classification	Stages of CC	Spectral data	LOOCV	DeepLabv3+ D-LinkNet	DSC = 0.95	-
[146]	2023	Treatment	Regression	Therapeutic dose and planning	CT images	-	U-Net	Score > 1%	-
[163]	2023	Diagnosis	Classification	Stages of CC	Clinical history	-	Stacking models	AUROC = 87%	-
[36]	2022	Diagnosis	Classification	Stages of CC	Colposcopy images	5	ResNet50	ACC = 81.3%	-
[37]	2022	Treatment	Regression	Therapeutic dose and planning	CT images	-	ResNet	DSC > 0.94	-
[32]	2022	Treatment	Image Segmentation	Segmentation of targets/OARs	MRI images	-	Inception ResNetv2	DSC = 0.72	-
[40]	2022	Treatment	Image Segmentation	Segmentation of targets/OARs	CT images	-	CNN	DSC > 0.70	-
[41]	2022	Diagnosis	Classification	Screening for CC	Cytology images	5	CNN	ACC = 95.4%	-
[42]	2022	Diagnosis	Classification	Screening for CC	Cytology images	-	YOLO ResNet	ACC = 90.5%	-
[43]	2022	Diagnosis	Classification	Screening for CC	Clinical history	5	Voting	ACC = 96.6%	-
[44]	2022	Diagnosis	Image Segmentation	Screening for CC	Colposcopy images	-	DeepLab V3+	ACC = 91.2%	-
[134]	2022	Prognosis	Regression	Survival	Histopathology images	-	CNN	AUC = 80%	-
[136]	2022	Prognosis	Regression	Cancer progression	MRI images PET images	5	CNN	AUC = 84%	-
[137]	2022	Prognosis	Classification	Cancer progression	Clinical history	-	MLP	AUC = 82%	-
[139]	2022	Diagnosis	Classification	Stages of CC	CT images	5	CNN	ACC > 60%	-
[140]	2022	Treatment	Image Segmentation	Segmentation of targets/OARs	CT images	-	CNN	DSC = 0.88	-
[141]	2022	Treatment	Image Segmentation	Delineation of the CTV	CT images	-	VN-Net	DSC = 0.81	-
[142]	2022	Diagnosis	Classification	Stages of CC	MRI images	5	CycleGAN	AUC = 89%	-
[143]	2022	Diagnosis	Classification	Stages of CC	Cytology images	-	CNN	SEN = 89%	-
[144]	2022	Treatment	Regression	Therapeutic dose and planning	Dose volume	-	U-Net	MAE = 2.4	Yes
[161]	2022	Treatment	Image Segmentation	Segmentation of targets/OARs	MRI images	-	CNN	Precision = 93%	-
[162]	2022	Diagnosis	Classification	Stages of CC	Colposcopy images	-	Dense-U-Net	ACC = 89%	Yes
[50]	2021	Diagnosis	Image Segmentation	Screening for CC	Cytology images	-	CNN	DSC = 0.92	-
[51]	2021	Diagnosis	Classification	Stages of CC	Cytology images	-	ResNet RNN CNN	SEN = 95.1%	Yes
[52]	2021	Diagnosis	Classification	Stages of CC	Cytology images	-	ResNet	ACC > 90%	Yes
[53]	2021	Diagnosis	Classification	Stages of CC	Colposcopy images	5	ResNet	AUC = 97%	-
[54]	2021	Diagnosis	Classification	HPV type	DNA	-	CNN	AUROC = 85%	Yes
[57]	2021	Treatment	Image Segmentation	Delineation of the CTV	CT images	3	U-Net CNN	DSC = 0.734	Yes
[59]	2021	Diagnosis	Image Segmentation	Screening for CC	Cytology images	10	YOLO v3	SEN = 92%	Yes
[60]	2021	Diagnosis	Classification	Screening for CC	Colposcopy images	-	CNN	ACC = 92%	-
[61]	2021	Prognosis	Classification	Cancer progression	MRI images	10	CNN	AUC = 91%	-
[63]	2021	Diagnosis	Classification	Screening for CC	Cytology images	5	CNN	ACC = 94%	-
[64]	2021	Treatment	Image Segmentation	Segmentation of targets/OARs	CT images	-	CNN	DSC = 0.85	Yes
[65]	2021	Diagnosis	Classification	Stages of CC	MRI images	-	XceptionNet	AUC = 93%	-
[66]	2021	Diagnosis	Classification	Screening for CC	Cytology images	5	CNN	AUC = 77%	-
[67]	2020	Treatment	Image Segmentation	Delineation of the CTV	CT images	-	CNN	DSC > 0.81	-
[68]	2020	Treatment	Image Segmentation	Therapeutic dose and planning	CT images	5	CNN	DSC > 0.82	-
[69]	2020	Treatment	Image Segmentation	Therapeutic dose and planning	CT images Dose volume	-	CNN	DVH = 0.73	-
[70]	2020	Diagnosis	Classification	Screening for CC	Colposcopy images	-	Faster R-CNN	AUC > 90%	-
[71]	2020	Diagnosis	Classification	Stages of CC	Cytology images	-	CNN	SEN = 100%	-
[72]	2020	Diagnosis	Classification	Stages of CC	Colposcopy images	10	Resnet	AUC = 78%	-
[73]	2020	Treatment	Image Segmentation	Segmentation of targets/OARs	CT images	-	CNN	DSC > 0.87	-
[75]	2020	Diagnosis	Image Segmentation	Stages of CC	Colposcopy images	-	CNN	ACC = 84%	-
[76]	2020	Diagnosis	Classification	Screening for CC	Colposcopy images	-	RetinaNet	AUC = 95%	-
[77]	2020	Prognosis	Image Segmentation	Cancer progression	MRI images	-	CNN	AUC = 93%	-
[79]	2020	Diagnosis	Classification	Screening for CC	Colposcopy images	-	CNN	ACC = 91%	-
[82]	2020	Diagnosis	Classification	Stages of CC	Cytology images	-	VGG-19	ACC = 95%	-
[83]	2020	Diagnosis	Classification	Stages of CC	MRI images Treatment plan	LOOCV	CNN	ACC = 94.3%	-
[85]	2020	Treatment	Image Segmentation	Therapeutic dose and planning	MRI images	5	UNet	SEN = 89%	-
[86]	2020	Treatment	Classification	Therapeutic dose and planning	Dose volume	-	CNN	*p* < 0.017	-
[87]	2020	Treatment	Image Segmentation	Segmentation of targets/OARs	CT images	-	UNet	DSC > 0.791	Yes
[88]	2019	Prognosis	Classification	Cancer progression	CT images	7	CNN	ACC = 89%	-
[89]	2019	Diagnosis	Classification	Stages of CC	Interferometric Measurements	-	CNN	SEN = 100%	-
[90]	2019	Diagnosis	Classification	Stages of CC	Spectral data	LOOCV	CNN	ACC = 100%	-
[22]	2019	Diagnosis	Classification	Stages of CC	Cytology images	-	CNN	ACC > 94%	-
[91]	2019	Diagnosis	Classification	Stages of CC	Cytology images	10	AGVFSM	ACC = 99%	-
[92]	2019	Diagnosis	Classification	Screening for CC	Colposcopy images	-	CNN	AUC = 91%	-
[93]	2019	Diagnosis	Classification	Stages of CC	CT images	-	CNN	ACC > 90%	-
[95]	2019	Treatment	Regression	Therapeutic dose and planning	Dose volume Histograms	-	Reinforcement learning	Score > 8.5%	Yes
[96]	2019	Diagnosis	Image Segmentation	Screening for CC	Cytology images	5	CNN	ACC = 91%	-
[97]	2019	Treatment	Image Segmentation	Segmentation of targets/OARs	CT images	5	CNN	DSC = 0.84	-
[98]	2019	Prognosis	Regression	Survival	Clinical history	5	FFNN	MAE = 29.3	-
[99]	2019	Diagnosis	Image Segmentation	Screening for CC	Cytology images	-	CNN	MAP = 0.936	-
[98]	2019	Prognosis	Regression	Survival	Clinical history	-	FFNN	MAE = 29.3	-
[102]	2018	Diagnosis	Classification	Stages of CC	Cytology images	-	CNN	Precision > 89%	Yes
[103]	2018	Diagnosis	Classification	Screening for CC	Colposcopy images	-	CNN	ACC = 100%	-
[155]	2018	Treatment	Image Segmentation	Segmentation of targets/OARs	CT images PET images	-	Graph cut	DSC = 0.83	-
[101]	2017	Treatment	Classification	Toxicity prediction in radiotherapy	CT images Treatment plan	10	VGG16	AUC = 89%	-
[107]	2015	Diagnosis	Classification	Screening for CC	Clinical history	-	NER	F1 = 67%	-
[110]	2015	Diagnosis	Image Segmentation	Stages of CC	Cytology images	-	GMM	DSC = 0.92	-

**Table 5:** Summary of ML and DL-based applications reported in the literature.

Ref.	Year	Clinical Application	Prediction Task	Target	Datasets	No. Folds For CV	Best Performance on Test Set		Externalval
							**Model**	**Metric**	
[160]	2024	Diagnosis	Classification	Stages of CC	Cytology images	-	ResNet152 LR	ACC = 98%	-
[184]	2024	Treatment	Image segmentation	Segmentation of targets/OARs	MRI images	-	SVM - RF ResNet50	ACC = 75%	-
[151]	2023	Diagnosis	Classification	Stages of CC	Cytology images	4	KNN ResNet50 ViT-S/16	ACC > 83%	-
[45]	2021	Diagnosis	Classification	Screening for CC	Clinical history	-	RF SNN	ACC = 93.6%	-
[48]	2021	Diagnosis	Image segmentation	Stages of CC	Histopathology images	5	U-Net SVM	ACC = 94.4%	-
[49]	2021	Diagnosis	Classification	Stages of CC	Histopatology images	10	CNN SVM	ACC = 97.4%	-
[169]	2021	Prognosis	Classification	Recurrence (Cancer progression)	DNA	10	SVM RNN	ACC = 92%	-

**Table 6:** Main computational challenges in cervical cancer using ML and DL models.

1. Data availability
2. Data leakage
3. Limited external validation
4. Limited evaluation of model performance
5. Complex data
6. Privacy issues
7. Lack of explainability
8. Lack of reproducibility

**Table 7:** Main challenges for clinical implementation.

1. Representativeness of clinical stages of CC in the training of deep learning-based models.
2. Privacy concerns and data security in health care.
3. Integration of AI with clinical workflows for real-time decision-making.
4. Ethical and regulatory considerations.
5. Public perspectives on using AI in their diagnoses and treatment decisions.

## Data Availability

No new data were created.

## Supplementary Materials

The following supporting information is available online at: https://www.mdpi.com/article/10.3390/diagnostics15121543/s1. Supplementary Table S1: Preferred Reporting Items for Systematic reviews and Meta-Analyses extension for Scoping Reviews (PRISMA-ScR) Checklist.

## Abbreviations

The following abbreviations are used in this manuscript:

ACC	Accuracy
AI	Artificial intelligence
AUC	Area Under (AUC) the Receiver Operating Characteristic (ROC) curve
CC	Cervical cancer
CLF	Classifier
CNN	Convolutional neural network
CT	Computed tomography
CTV	Clinical target volume
DCNN	Deep convolutional neural networks
DL	Deep learning
DRS	Diffuse reflectance spectroscopy
DT	Decision tree
FS	Feature selection
GPC	Gaussian process classifier
GMM	Gaussian mixture model
HPV	Human Papilloma Virus
KNN	K-Nearest neighbors
LDA	Linear discriminant analysis
LR	Logistic regression
Mask R-CNN	Mask regional CNN
MGI	Magnetic resonance imaging
ML	Machine Learning
MLP	Multi-layer perceptron
NB	Naive Bayes
OAR	Organ at risk
PCA	Principal component analysis
RF	Random forest
RNN	Recurrent neural network
SVM	Support vector machine
ViT	Vision transformer
